# In Tuber Biocontrol of Potato Late Blight by a Collection of Phenazine-1-Carboxylic Acid-Producing *Pseudomonas* spp.

**DOI:** 10.3390/microorganisms9122525

**Published:** 2021-12-07

**Authors:** Geneviève Léger, Amy Novinscak, Adrien Biessy, Simon Lamarre, Martin Filion

**Affiliations:** 1 Biology Department, Université de Moncton, Moncton, NB E1A 3E9, Canada; pgl3061@umoncton.ca (G.L.); simon.lamarre@umoncton.ca (S.L.); 2 Agassiz Research and Development Centre, Agriculture and Agri-Food Canada, Agassiz, BC V0M 1A2, Canada; amy.novinscak@agr.gc.ca; 3 Saint-Jean-sur-Richelieu Research and Development Centre, Agriculture and Agri-Food Canada, Saint-Jean-sur-Richelieu, QC J3B 7B5, Canada; adrien.biessy@agr.gc.ca

**Keywords:** *Pseudomonas*, *Phytophthora infestans*, biocontrol, phenazine, *Solanum tuberosum*

## Abstract

Phenazine-1-carboxylic acid (PCA) produced by plant-beneficial *Pseudomonas* spp. is an antibiotic with antagonistic activities against *Phytophthora infestans*, the causal agent of potato late blight. In this study, a collection of 23 different PCA-producing *Pseudomonas* spp. was confronted with *P. infestans* in potato tuber bioassays to further understand the interaction existing between biocontrol activity and PCA production. Overall, the 23 strains exhibited different levels of biocontrol activity. In general, *P. orientalis* and *P. yamanorum* strains showed strong disease reduction, while *P. synxantha* strains could not effectively inhibit the pathogen’s growth. No correlation was found between the quantities of PCA produced and biocontrol activity, suggesting that PCA cannot alone explain *P. infestans*’ growth inhibition by phenazine-producing pseudomonads. Other genetic determinants potentially involved in the biocontrol of *P. infestans* were identified through genome mining in strains displaying strong biocontrol activity, including siderophores, cyclic lipopeptides and non-ribosomal peptide synthase and polyketide synthase hybrid clusters. This study represents a step forward towards better understanding the biocontrol mechanisms of phenazine-producing *Pseudomonas* spp. against potato late blight.

## 1. Introduction

More than 150 years after the Irish Potato Famine, potato late blight caused by the oomycete *Phytophthora infestans* still poses a threat to potato production worldwide. Despite efforts invested in breeding programs and genetic engineering to create late blight resistant cultivars, limited success has been obtained. Current control strategies focus mostly on prevention and still heavily rely on the use of chemical pesticides [1,2]. Most potato cultivars of commercial interest are susceptible to late blight, and *P. infestans* strains are becoming more aggressive and resistant to chemical pesticides such as metalaxyl, a commonly used systemic phenylamide fungicide [3,4]. In the USA and Canada, *P. infestans* strains US-8 (A2 mating type), US-23 (A1 mating type) and US-24 (A1 mating type) are the dominant genotypes. US-8 is metalaxyl resistant, US-24 is tolerant, and US-23 is sensitive, but shows increasing resistance with prolonged exposure [5,6]. Therefore, alternative methods are sought to control late blight of potato, such as biocontrol.

Biocontrol is defined as the use of a living organism to reduce the symptoms of a disease, and involves an interaction between a plant, a pathogen, and a biocontrol agent [7]. Bacteria belonging to different taxonomical groups have been identified as promising biocontrol agents, with the most studied ones belonging to the *Pseudomonas* and *Bacillus* genera. These bacteria can also often promote plant growth and reduce disease symptoms by using several mechanisms, such as improving plant nutrition, stimulating the plant’s natural resistance mechanisms, competing with pathogens, or producing antibiotics capable of inhibiting the metabolism or growth of pathogens [7,8].

*Pseudomonas* spp. are Gram-negative bacteria that have been studied as biocontrol agents against numerous plant pathogens. Several strains of *Pseudomonas* spp. are of interest for biocontrol because they actively compete with other microorganisms, they have a versatile metabolism, and they produce several antibiotics with antagonistic effects against phytopathogens, such as phenazines [9]. Phenazines are nitrogenous heterocyclic compounds that act as reducing agents, causing the uncoupling of oxidative phosphorylation and reducing the activities of reactive oxygen species (ROS) detoxification enzymes, thus leading to the accumulation of toxic molecules inside the targeted cell [10,11,12]. They also contribute to the inhibition of the carbohydrate metabolism and reduce nutrient absorption, therefore decreasing energy production [13]. In addition to their antifungal and antibacterial properties, phenazines also allow *Pseudomonas* spp. that produce them to better compete with the microflora of the rhizosphere, making them suitable candidates as biocontrol agents [14].

In *Pseudomonas* spp., phenazine production is mediated by a seven-gene operon, *phzABCDEFG*. In addition, other genes may also be involved in the conversion of phenazine-1-carboxylic acid (PCA), the base molecule, into other phenazine derivatives such as phenazine-1-carboxamide (PCN), 2-hydroxyphenazine-1-carboxylic acid (2-OH-PCA) and 2-hydroxyphenazine (2-OH-PHZ) [11,15]. Numerous studies have shown that reduction in pathogen growth and disease development is correlated with production of phenazine compounds. For example, PCA-producing *Pseudomonas synxantha* (formerly *P. fluorescens*) 2-79 was found effective against *Gaeumannomyces graminis* var. *tritici* causing take-all of wheat [16], PCN-producing *Pseudomonas chlororaphis* subsp. *aurantiaca* Pcho10 reduced the growth of *Furasium graminearum* responsible for Fusarium head blight [17], and PCA-producing *Pseudomonas synxantha* (formerly *P. fluorescens*) LBUM223 reduced potato common scab symptoms caused by *Streptomyces scabies* under controlled and field conditions [18,19], to name a few.

To date, some studies have focused specifically on the biocontrol of potato late blight by phenazine-producing *Pseudomonas* spp. The work of Morrison et al. [20] demonstrated that *P. infestans*’ growth was repressed by *Pseudomonas yamanorum* (formerly *P. fluorescens*) LBUM636 under *in planta* and soil conditions due to the inhibitory activity of PCA. *Pseudomonas synxantha* LBUM223 has also been studied for its biocontrol activity against *P. infestans*, and it was reported that wildtype PCA-producing LBUM223 significantly inhibited *P. infestans*’ growth in in vitro confrontational assays compared to a PCA-deficient isogenic mutant, in addition to altering its transcriptome [21]. De Vrieze et al. [22] also demonstrated that, compared to non-phenazine producers, 2-OH-PCA- and PCA-producing *Pseudomonas chlororaphis* R47 greatly inhibited mycelial growth of multiple *Phytophthora infestans* strains.

Biessy et al. [23] recently published a paper describing and comparing the genomes of a collection of 63 strains of phenazine-producing *Pseudomonas* spp., representative of the worldwide diversity of phenazine-producing *Pseudomonas* spp. The inhibitory activity of these 63 *Pseudomonas* spp. strains against three potato pathogens—*P. infestans*, *S. scabies* and *Verticillium dahliae*—was also evaluated in in vitro confrontational assays [24]. To further improve our knowledge of biocontrol and the role played by phenazines, we carried out *in planta* antagonisitic assays in potato tubers against *P. infestans* using a subset of 23 *Pseudomonas* spp. strains from this collection that produce only PCA (and no other phenazine derivatives). More specifically, the aims of this study were to: (i) evaluate the biocontrol activity of a collection of 23 PCA-producing *Pseudomonas* spp. strains against late blight in potato tuber assays; (ii) quantify their PCA production alone and in combination with *P. infestans* in potato tuber assays; and (iii) identify, using genome mining tools, other potential genetic determinants in these strains involved in the biocontrol of *P. infestans*.

## 2. Materials and Methods

### 2.1. Bacterial Strains, Growth Conditions, and Preparation of the Inocula

**Figure 1:**
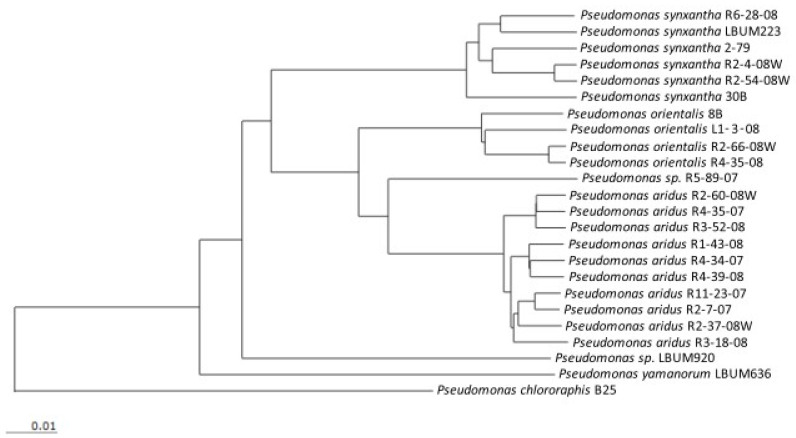
Neighbor-joining phylogeny of 23 PCA-producing *Pseudomonas* spp. strains. The phylogenetic tree was generated by EDGAR [25] from a concatenated alignment of 2843 genes. *P. chlororaphis* B25 was used as an outgroup.

The 23 PCA-producing *Pseudomonas* strains used in this study (see Table 1) belong to the *Pseudomonas fluorescens* species complex and have already been described in a previous study [23]. A phylogenetic tree was generated by EDGAR [25] from a concatenated alignment of 2843 genes found among these 23 strains and is shown in Figure 1. Bacterial strains were grown in King’s B broth [26] at 25 °C with constant shaking for 48 h. Populations were measured using a spectrophotometer (λ = 600 nm) and previously determined standard curves. Each strain was diluted to 3.5 × 10^8^ CFU/mL using sterile distilled water.

### 2.2. Pathogen Strain, Growth Conditions, and Preparation of the Inoculum

*Phytophthora infestans* (US-8 strain) was grown on V8 agar (200 mL V8 juice, 3 g CaCO_3_, 15 g agar and 800 mL water) at 20 °C for general propagation, and 10% unclarified V8 agar [33] at 15 °C for sporangia production. Sporangia inoculum was prepared according to Morrison et al. [20] and diluted to 3 × 10^3^ sporangia/mL using sterile tap water.

### 2.3. Potato Tuber Antagonistic Assays with Pseudomonas spp.

The biocontrol capabilities of the 23 *Pseudomonas* spp. strains against *P. infestans* were ascertained in *in tuber* confrontational assays using a simplified potato tuber (Russet cultivar) system, as described by Morrison et al. [20]. A 1.5 cm deep hole was carved into each tuber using a 6 mm cork borer. Twenty μL of *P. infestans* sporangial inoculum was added to the hole for all treatments requiring it and allowed to set for 1 h. Twenty μL of bacterial inoculum (for confrontation assays) or sterile tap water (for pathogen-only and bacteria-only controls) was then added to all treatments requiring it. Negative controls received 40 μL of sterile tap water instead of bacterial or pathogen sporangial inocula. In total, 48 treatments were used: 23 treatments with each *Pseudomonas* strain alone, 23 treatments with a combination of each *Pseudomonas* + *P. infestans*, 1 treatment with *P. infestans* alone, and 1 negative control (water) treatment. Five replicates per treatment were used and the whole experiment was repeated twice. Tubers were incubated at 15 °C in the dark for 3 weeks, then cut in half and photographed.

### 2.4. PCA Extractions

Potato samples were retrieved using a 10 mm cork borer around the inoculation hole on each potato half, and kept at 4 °C until PCA extractions were performed. The protocol for sample preparation was based on Brazinskiene et al. [34], with modifications. The potato samples were ground fresh, then 1 mL of acetonitrile and a 3 mm tungsten carbide bead were added to the samples and ground with a TissueLyser II (Qiagen, Hilden, Germany), at 30 Hz for 3 min. After centrifugation, the supernatants were filtered through a 0.20 μm pore nylon membrane and diluted in 4 mL water + 0.1% formic acid (FA). Samples were then filtered through a Bond Elut C18 column (Agilent Technologies, Santa Clara, CA, USA), and retrieved with 250 μL acetonitrile. Concentrated PCA samples were then diluted with water + 0.1% FA in a 1:4 factor.

PCA standards were also prepared and inoculated in potato tubers for method optimisation and validation. Three PCA standards were prepared using purified PCA dilutions of known concentrations (5 × 10^4^, 1 × 10^5^ and 5 × 10^5^ ng/mL), and 20 μL were inoculated in potato tubers. PCA extractions were performed as described above.

### 2.5. PCA Quantification Using HPLC Analyses

In order to quantify PCA, the samples were analysed using high performance liquid chromatography (HPLC) (Agilent 1100 Series HPLC Value System, Agilent Technologies). Analyses were performed using a Synergi™ 4 μm Hydro-RP 80 Å, 100 × 2 mm column (Phenomenex, Torrance, CA, USA). For the analysis, 50 μL of PCA solution were injected and tested at a mobile phase flow rate of 0.4 mL/min. The following gradient system was used: solvent A—0.1% FA in water, solvent B—0.1% FA in acetonitrile; 0 min—95% A and 5% B, 15 min—0% A and 100% B, 15.01 min—95% A and 5% B.

PCA concentrations were calculated using the software OpenLAB CDS ChemStation Edition version A0.2.13 (Agilent Technologies). The PCA amounts computed by the software were corrected for the dilution factor and the potato samples’ mass.

### 2.6. Potato Tuber Confrontational Assays with Exogenous Applied PCA

In another experiment following the same protocol as described above, *P. infestans* was confronted with 5 different concentrations of purified PCA: 0, 5 × 10^3^, 1 × 10^4^, 5 × 10^4^ and 1 × 10^5^ ng of PCA per mL. The PCA dilutions were prepared using a stock solution of 0.01 g purified PCA (Ryan Scientific Inc, Mt Pleasant, SC, USA) diluted in 1 mL TRIS 1M (pH 10), then further diluted with TRIS 1 M to concentrations of 5 × 10^3^, 1 × 10^4^, 5 × 10^4^ and 1 × 10^5^ ng/mL. Twenty μL of PCA dilutions and 20 μL of *P. infestans* sporangial inoculum were applied to potato tubers (*n* = 10). Negative controls received 40 μL of water, and pathogen-only controls received 20 μL of *P. infestans* inoculum and 20 μL of water. Tubers were incubated for 3 weeks, then cut in half and photographed.

### 2.7. Statistical Analyses

The software RStudio version 1.4.1106 (RStudio Inc, Boston, MA, USA) was used to perform statistical analyses. Correlations between phenazine production and biocontrol activity for both conditions (*Pseudomonas* spp. alone or in confrontation with *P. infestans*) were calculated using Kendall rank correlation coefficient τ (R function cor.test (x, y, method = “kendall”)).

### 2.8. Identification of Other Genetic Determinants of Biocontrol Interest

Based on available genome sequences, we identified traits only associated with strains displaying strong *in planta* biocontrol activity, that may therefore be involved in the biocontrol of *P. infestans*. The *Pseudomonas* genome database was consulted to retrieve genes of interest used thereafter as baits to identify putative orthologs in the genomes under study. Most of the baits described in Biessy et al. [23] were used in this study. Secondary metabolites production clusters were identified using antiSMASH [35,36]. Putative antibacterial proteins were identified by whole genome Pfam analysis using CLC Genomic Work-bench 9.0 and analysed subsequently using the InterPro website [37].

## 3. Results

### 3.1. Potato Tuber Antagonistic Assays

**Figure 2:**
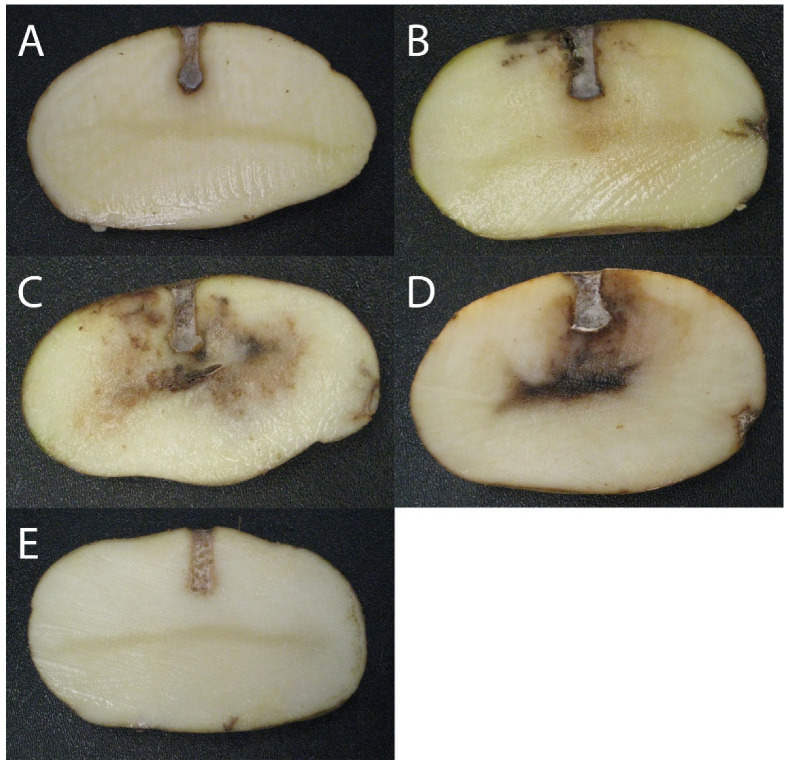
Photos of potato tuber antagonistic assays with *P. infestans* and *Pseudomonas* spp. representative of the different symptom classes observed. Confrontations with (**A**) *P. yamanorum* LBUM636 (strong biocontrol); (**B**) *P. aridus* R4-39-08 (intermediate biocontrol); (**C**) *P. synxantha* R2-4-08W (weak biocontrol); (**D**) Positive control (*P. infestans*); (**E**) Negative control (water).

The biocontrol activity of each of the 23 *Pseudomonas* spp. strains against *P. infestans* was evaluated using potato tuber antagonistic assays. The results are presented in Table 2. Each tuber was evaluated for the severity of late blight symptoms on the exposed side of the cut tuber compared to the controls, and each strain was categorized among three groups according to the results’ average: (1) weak biocontrol activity, (2) intermediate biocontrol activity, and (3) strong biocontrol activity. Photographs of the disease suppression observed for selected *Pseudomonas* spp. strains representative of each biocontrol group are shown in Figure 2, and representative photos of inoculation assays for all 23 *Pseudomonas* spp. strains can be seen in Figure S1.

Seven *Pseudomonas* spp. were categorized in the “strong biocontrol activity” group, where no late blight symptoms were detected; twelve *Pseudomonas* spp. were categorized in the “intermediate biocontrol activity” group, where mostly only light brown rot was observed around the tuber’s inoculation hole; and finally four strains were categorized in the “weak biocontrol activity” group, where dark brown rot around the tuber’s inoculation hole and upper surface could be seen, comparable to the disease severity observed in pathogen-only controls. No clear taxonomic segregation between the 3 biocontrol groups was observed, but there seems to be a general pattern in each taxonomic group relative to late blight disease suppression: in general, the *P. orientalis* group, as well as the three strains not categorized in either the *P. orientalis*, *P. synxantha* or *P. aridus* groups showed higher inhibition capabilities, while strains belonging to the *P. synxantha* group were not as efficient at inhibiting *P. infestans*’ growth. Most strains belonging to the *P. aridus* group showed partial late blight control.

**Figure 3:**
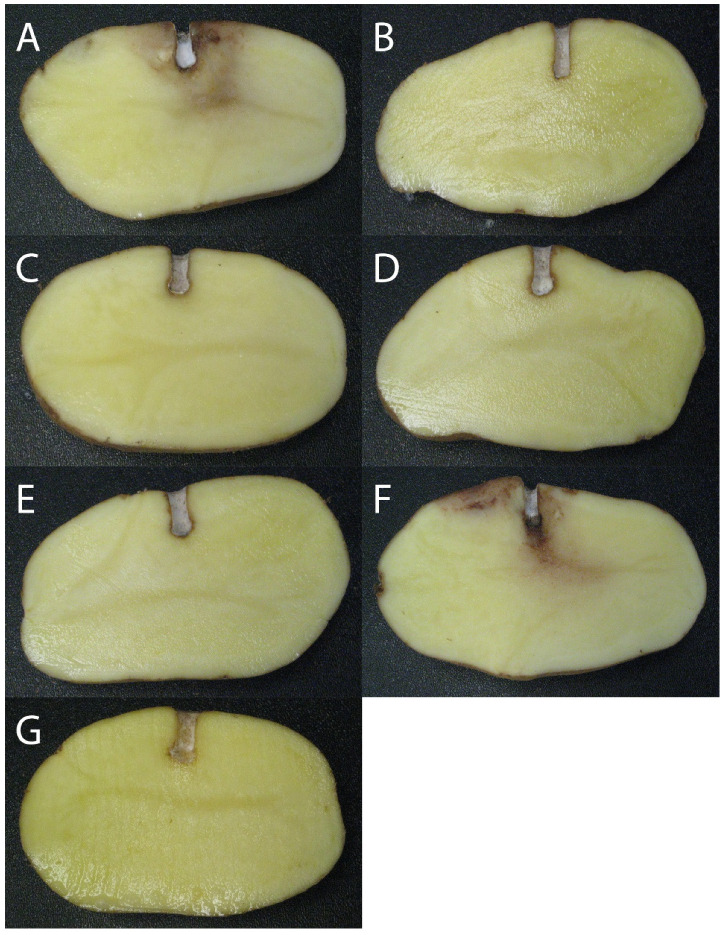
Photos of potato tuber antagonistic assays with *P. infestans* using different exogenous PCA concentrations diluted in TRIS 1M.(A) 0 ng/mL; (**B**) 5 × 10^3^ ng/mL; (**C**) 1 × 10^4^ ng/mL; (**D**) 5 × 10^4^ ng/mL; (**E**) 1 × 10^5^ ng/mL; (**F**) Positive control (*P. infestans*); (**G**) Negative control (water).

Photographs of inoculation assays using purified PCA dilutions are shown in Figure 3. No symptoms were observed on all the tubers that received PCA treatment, regardless of the concentration applied.

### 3.2. PCA Quantification Using HPLC

**Figure 4:**
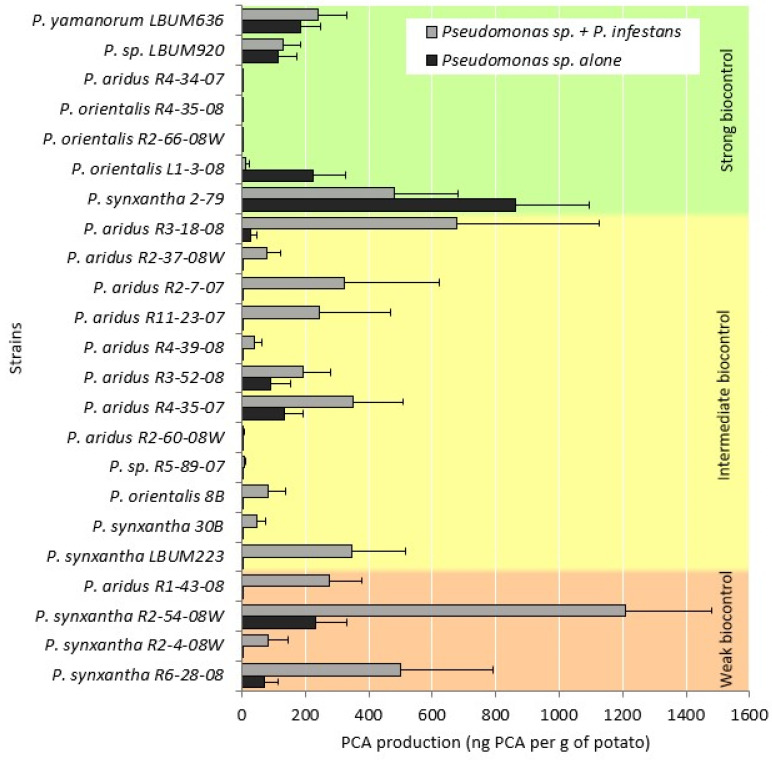
PCA production by 23 *Pseudomonas* spp. strains alone and in the presence of *P. infestans* in potato tuber assays. Bars represent standard errors.

In order to verify if the ability to control *P. infestans* was related or not to phenazine production, PCA concentrations in potato tubers assays were measured by HPLC. Production of PCA by each of the 23 *Pseudomonas* spp. strains, alone and in confrontation with *P. infestans*, is shown in Figure 4. Retention time (RT) varied slightly between experiments, as the runs’ averages ranged from 7.054 min to 7.128 min, but samples of known PCA concentrations were analyzed with each experiment and used as standards to establish a standard curve linked to each run. Samples with a retention time varying more than ±0.05 min of the run’s PCA standards’ RT were excluded from analyses.

No PCA was detected in 17 of the 46 experimental treatments (14 that received only *Pseudomonas* sp. inocula, and three that received both bacterial and sporangial inoculations; see Figure 4). Three out of the seven strains belonging to the “strong biocontrol activity” group produced no PCA under our conditions, while the strain producing the highest concentration of PCA while in confrontation with *P. infestans* could not inhibit late blight symptoms. Kendall’s correlation test revealed no significant correlation between biocontrol activity and phenazine production, in the presence and in the absence of *P. infestans* (*p*-values > 0.05).

### 3.3. Identification of Other Genetic Determinants of Biocontrol Interest

**Figure 5:**
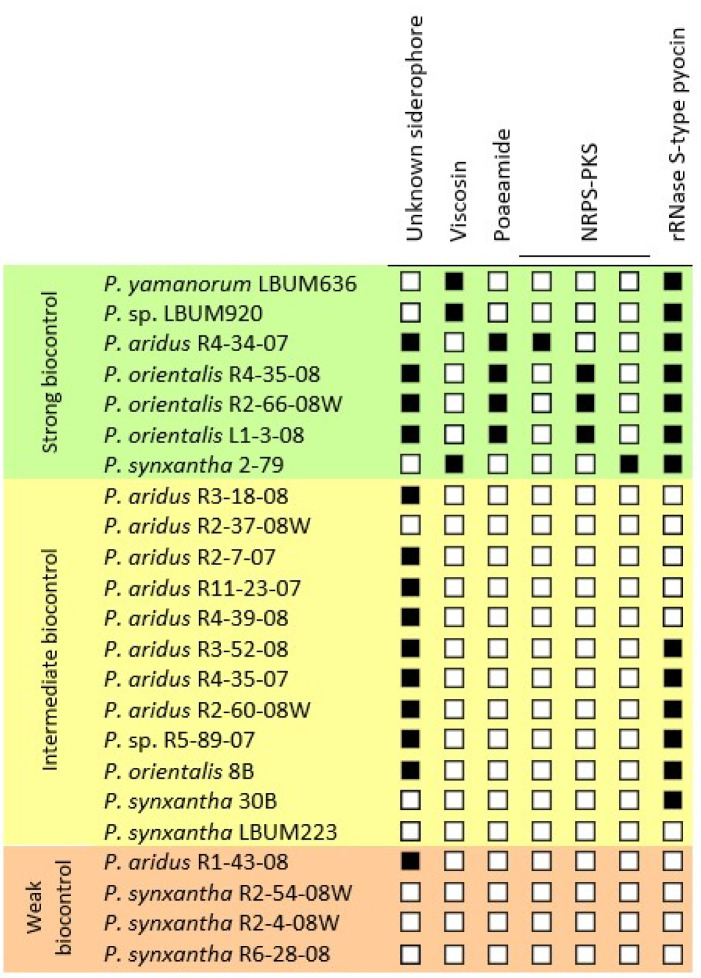
Potential genes and gene clusters involved in biocontrol abilities of 23 PCA-producing *Pseudomonas* spp. strains. The presence of a gene/cluster is symbolized by a black square, and the absence is indicated by a white square. NRPS: Non ribosomal peptide synthase; PKS: Polyketide synthase.

Several phytobeneficial traits potentially involved in the biocontrol of late blight of potato were found among the *Pseudomonas* spp. under study. Traits of interest were found in the genome of strains that displayed strong biocontrol activities but were absent from the genome of strains belonging to the weak biocontrol group. We identified biosynthetic clusters associated with siderophores, viscosins, poaeamides, non-ribosomal peptide synthases (NRPS) and polyketide synthases (PKS), and rRNase S-type pyocins (Figure 5).

## 4. Discussion

The potato tuber confrontational assays clearly showed that despite their ability to produce PCA, not all *Pseudomonas* spp. strains used in this study were able to effectively control potato late blight under our conditions. Despite most strains being taxonomically closely related, there is a high phenotypical variation among PCA-producing *Pseudomonas* spp. As a whole, the *P. synxantha* group seems to be less efficient at reducing late blight symptoms than the other *Pseudomonas* species under study, although there is a high variability observed among this group, with for example *P. synxantha* 2-79 being able to completely inhibit late blight symptoms, and *P. synxantha* R2-4-08W, R2-54-08W and R6-28-08 showing only weak symptoms reduction. The *P. orientalis* group generally shows strong biocontrol activity, but inoculations with each of the strains belonging to this species created a bigger hole than the original inoculation hole made by the 6 mm cork borer (see Figure S1). This happened with all 3 *P. orientalis* strains who displayed strong biocontrol activity, and to a lesser extent with *P. orientalis* 8B (intermediate biocontrol). This suggests that although these strains are able to completely inhibit late blight symptoms, they can also cause harm to the potato tuber, which could potentially interfere with their biocontrol activity under commercial conditions. On the other hand, most strains belonging to the *P. aridus* group displayed moderate disease symptoms reduction, with the exception of R1-43-08 that could not inhibit *P. infestans*’ growth, and R4-34-07 that displayed strong biocontrol activity. Regarding the 3 other strains that do not belong to either the *synxantha*, *aridus* and *orientalis* species, *P.* sp. R5-89-07 moderately inhibited *P. infestans*’ growth, while *P. yamanorum* LBUM636 and *P.* sp. LBUM920 completely inhibited late blight symptoms. *P. yamanorum* LBUM636 was also previously studied in Morrison et al. [20] in potato tuber antagonistic assays, and similar results were obtained here.

PCA concentrations measured by HPLC revealed no correlation between PCA production and biocontrol ability, hence suggesting that the amount of phenazine produced does not significantly affect biocontrol abilities. A striking feature that can be seen in Figure 4 is that four out of seven *Pseudomonas* spp. that display strong biocontrol activities produce no or very little PCA while in confrontation with *P. infestans*. Three of them belong to the *P. orientalis* group, while the other belongs to the *P. aridus* group. Despite the absence of PCA compounds in the analyzed samples, those *Pseudomonas* strains were able to completely inhibit *P. infestans*’ growth. On the other hand, two strains used in our study, *P. synxantha* R2-54-08W and R6-28-08, produced considerable amount of PCA (1st and 3rd highest producer, respectively) while in confrontation with *P. infestans*, but could not effectively inhibit *P. infestans*’ growth. Taken together, these results support the hypothesis that PCA by itself does not explain late blight symptoms reduction, and there are likely other determinants involved in the biocontrol of potato late blight by phenazine-producing *Pseudomonas* spp., either acting independently or in combination with phenazines.

Other studies also support this hypothesis. De Vrieze et al. [38] sequenced and compared the genomes of nine *Pseudomonas* spp. strains displaying inhibitory activities against *P. infestans*. Only one strain, *P. chlororaphis* R47 produces phenazines (PCA and 2-OH-PCA), therefore suggesting that phenazines are not the sole determinants of late blight biocontrol, since non-phenazine producing *Pseudomonas* spp. strains could also alter *P. infestans*’ growth. Likewise, Biessy et al. [24] characterized the inhibitory activities of 63 phenazine-producing *Pseudomonas* spp. against *P. infestans* in in vitro assays, and also measured under these conditions phenazine production by 13 strains of interest. They concluded that phenazine production, regardless of the phenazine compound, was not correlated with inhibition capabilities. Hence, phenazines cannot be considered as the only and/or main determinants in the biocontrol of potato late blight by phenazine-producing pseudomonads.

The antagonistic assays preformed in this study using purified PCA showed no symptoms on all tubers that received phenazine treatments. We chose four concentrations (5 × 10^3^, 1 × 10^4^, 5 × 10^4^ and 1 × 10^5^ ng of PCA per mL) based on the amount of PCA detected by HPLC in the tuber confrontational assays using bacterial and sporangial inoculations. Based on the purified PCA assays results, 5 × 10^3^ ng of purified PCA per mL were enough to completely inhibit late blight symptoms; however, we detected higher quantities in some antagonistical assays samples that showed weak biocontrol activity, for example *P. synxantha* R2-54-08W that produced nearly 12 times that amount but could not inhibit *P. infestans* growth. In this case, either PCA was not effective, was rendered inactive, or might have been produced after the establishment of late blight infection, and therefore was not able to protect the potato tuber. PCA is a secondary metabolite that is in part regulated by population density [10]; the amount of time it took for the *Pseudomonas* sp. population to produce such a high quantity of PCA might explain why *P. infestans* was able to efficiently infect the tubers. Since purified PCA was applied at nearly the same time as the *P. infestans* sporangial inoculum in the confrontational assays, *P. infestans* would have been more vulnerable to the inhibitory activity of phenazines, and therefore might not have been able to infect the tuber. It would be interesting to see if allowing the *Pseudomonas* spp. population to grow before inoculating *P. infestans* might improve biocontrol activity, considering that in agricultural use, the biocontrol agent would most likely be inoculated before the appearance and development of the disease.

Although all 23 *Pseudomonas* spp. strains used in this study harbor the phenazine operon [23], we could not detect PCA in 14 of the treatments which received only the bacterial inoculum and no *P. infestans* sporangia, and in three of the treatments also infected with *P. infestans*. This can be explained by the fact that phenazines are secondary metabolites, whose production represents a considerable cost for the cell [10]. Phenazines are also involved in colonization and competitiveness [14,39], but because there were no other microorganisms inoculated inside the potato tubers in the *Pseudomonas* spp. only treatments, phenazine production might not be necessary under these conditions. As supported by Figure 4 and the correlation tests, the presence of *P. infestans* seemed to stimulate PCA production in most strains, but its concentration did not significantly affect disease suppression. Another explanation for the absence of PCA detected in some of the samples could be that population densities in certain treatments were not high enough to trigger PCA production. One of the main regulators of phenazine production in pseudomonads is quorum sensing, a mechanism dependent on population density [11]. Although we inoculated the same concentration of bacteria inside potato tubers, some strains may not have been able to grow to population levels high enough to trigger detectable PCA concentrations. Using HPLC, we could not determine if the absence of PCA was due to the *Pseudomonas* spp. inability to produce PCA under our conditions, as it has already been established that PCA production by pseudomonads can be influenced by environmental conditions [11], or if the bacteria could not survive the 3 weeks incubation period inside the potato tuber. However, as three out of the seven strains belonging to the “strong biocontrol activity” group did not produce detectable amounts of PCA but displayed strong reduction in late blight symptoms, it suggests that these strains survived well enough to compete with *P. infestans* and inhibit its growth. Also, the HPLC system could not account for PCA degradation over time, which could be another explanation for the lack of PCA detection in many samples. However, the absence of PCA in samples displaying no disease symptoms supports the hypothesis that PCA produced by *Pseudomonas* spp. used in this study may not be the main determinant involved in the biocontrol of late blight of potato.

Based on available genome sequences of the 23 strains used in this study, we looked for possible additional contributors to the biocontrol of potato late blight by phenazine-producing pseudomonads (Figure 5) [23]. In addition to PCA, all 10 members of the *P. aridus* group, all four members of the *P. orientalis* group, as well as *P.* sp. R5-89-07 also possess a putative siderophore biosynthesis cluster. Siderophores are iron-chelating compounds involved in the transport of iron through the cell membrane of bacteria. Plant disease suppression have been linked to siderophore production by fluorescent pseudomonads (e.g., pyoverdine), as the resulting ferric-siderophore complex depletes the available iron in soil, and cannot be utilised by competing microorganisms such as phytopathogens [40,41]. The fact that *P. synxantha* strains, the phylogenetic group in our study that is less effective at controlling *P. infestans*, cannot produce this putative siderophore, suggests that it could be involved in the biocontrol of potato late blight.

Three *Pseudomonas* spp. under study harbour a viscosin biosynthetic cluster (Figure 5) [23]: *P. synxantha* 2-79, *P. yamanorum* LBUM636 and *P.* sp. LBUM920. Viscosins are cyclic lipopeptides whose inhibitory activity against *P. infestans* and other oomycetes has already been studied (reviewed in Raaijmakers et al. [42]). In particular, viscosins stimulate the induction of zoospores encystment, thus reducing the infectious capabilities of phytopathogens [43]. Interestingly, these three strains showed strong biocontrol activity against *P. infestans* under our conditions, thus making viscosin a possible contributor to the biocontrol of potato late blight.

The four strains belonging to the *P. orientalis* group harbour a poaeamide biosynthetic cluster, another cyclic lipopeptide (Figure 5) [23]. In addition to zoosporicidal activity, poaeamides produced by *Pseudomonas* spp. favor swarming mobility and root colonisation [44]. Considering that the *P. orientalis* group shows strong biocontrol activity against *P. infestans*, the fact that only this group possess this cluster might be an indication of its possible involvement in potato late blight biocontrol.

Non ribosomal peptide synthase (NRPS) and polyketide synthase (PKS) are large multi-functional enzymes that produce numerous compounds associated with biocontrol, such as pyoverdine and 2,4-diacetylphloroglucinol (DAPG) [45]. Three putative NRPS-PKS hybrid clusters were only found in the genomes of *Pseudomonas* spp. that completely inhibited *P. infestans* (Figure 5) [23]: one was found in *P. aridus* R4-34-07, one in *P. synxantha* 2-79, and one in the 3 *P. orientalis* belonging to the strong biocontrol group. The fact that these putative NRPS-PKS clusters are only present among the most effective strains of each taxonomical group suggests a potential implication in the biocontrol of late blight of potato.

We also identified genes encoding a S-type pyocin with a cytotoxic rRNase domain (Pfam PF09000) among a dozen strains displaying strong and intermediate biocontrol activity (Figure 5) [23]. S-type pyocins are bacteriocins produced by some *Pseudomonas* species that targets and kills bacteria of the same or closely related species. Studies have mostly focused on pyocins produced by *P. aeruginosa*, a human opportunistic pathogen, but rRNase pyocins have been found in *Pseudomonas* spp. isolated from soil and plant environment, and their exact role in biocontrol has yet to be determined [46,47]. They may for example be important for controlling pathogen-helper or disease-enhancing bacteria, but this would need to be further investigated.

Clearly, further studies will be required to validate the implication of these potential biocontrol traits against potato late blight caused by *P. infestans*. The principal aim of this study was to narrow down the interaction between biocontrol activity and PCA production, using a simplified potato tuber system. Further research involving plants grown in controlled settings and field experiments will be necessary to improve our understanding of potato late blight biocontrol by phenazine-producing pseudomonads. Also, in this study we used *P. infestans* strain US-8, an A2 mating type. In addition to US-8, strains US-23 and US-24, both belonging to the A1 mating type, are among the most dominating *P. infestans* strains found in Canada and the USA. It would be interesting to confront the 23 *Pseudomonas* spp. strains used in this study against those 2 other *P. infestans* strains in order to compare their biocontrol abilities with those against the US-8 strain. As this study shows potential commercial application, a biocontrol agent that has a broad range against multiple *P. infestans* populations is an important factor to consider.

## 5. Conclusions

In conclusion, we have compared the biocontrol activity of 23 PCA-producing *Pseudomonas* spp. strains in potato tubers antagonisitic assays against *P. infestans*, the causative agent of potato late blight. The results showed high variability in disease symptoms’ reduction among those 23 strains, even though the *Pseudomonas* spp. strains all harbour the phenazine biosynthetic operon. Furthermore, we quantified PCA concentrations and found no correlation between PCA production and biocontrol activity. Those results support our hypothesis that other key determinants are involved in the biocontrol of potato late blight, either acting alone or in combination with phenazines. Among these determinants found in strains displaying strong biocontrol activity, siderophores, cyclic lipopeptides and NRPS-PKS hybrid clusters have been identified in this study as potential key biocontrol contributors. Transcriptome analyses and reverse genetic approaches will be needed to identify and validate those other biocontrol traits and further our understanding of the biocontrol mechanisms of phenazine-producing *Pseudomonas* spp. This study represents a step forward in researching and developing an environmentally friendly and sustainable biocontrol agent for commercial use against potato late blight.

## Figures and Tables

**Table 1:** *Pseudomonas* spp. strains used in this study.

Strain	Origin	Genome Accession Number	Reference or Source
*P. synxantha* R6-28-08	Wheat, USA	CP027756	Parejko et al. [27]
*P. synxantha* LBUM223	Strawberry, Canada	CP011117	Roquigny et al. [28]
*P. synxantha* 2-79	Wheat, USA	CP027755	Weller & Cook [29]
*P. synxantha* R2-4-08W	Wheat, USA	CP027757	Parejko et al. [27]
*P. synxantha* R2-54-08W	Wheat, USA	CP027758	Parejko et al. [27]
*P. synxantha* 30B	Wheat, Iran	CP027754	Shirzad et al. [30]
*P. orientalis* 8B	Wheat, Iran	CP027723	Shirzad et al. [30]
*P. orientalis* L1-3-08	Wheat, USA	CP027724	Parejko et al. [27]
*P. orientalis* R2-66-08W	Wheat, USA	CP027725	Parejko et al. [27]
*P. orientalis* R4-35-08	Wheat, USA	CP027726	Parejko et al. [27]
*P.* sp. R5-89-07	Wheat, USA	CP027727	Parejko et al. [27]
*P. aridus* R2-60-08W	Wheat, USA	CP027731	Parejko et al. [27]
*P. aridus* R4-35-07	Wheat, USA	CP027732	Mavrodi et al. [31]
*P. aridus* R3-52-08	Wheat, USA	CP027730	Parejko et al. [27]
*P. aridus* R1-43-08	Wheat, USA	CP027734	Parejko et al. [27]
*P. aridus* R4-34-07	Wheat, USA	CP027760	Mavrodi et al. [31]
*P. aridus* R4-39-08	Wheat, USA	CP027733	Parejko et al. [27]
*P. aridus* R11-23-07	Wheat, USA	CP027761	Mavrodi et al. [31]
*P. aridus* R2-7-07	Wheat, USA	CP027759	Mavrodi et al. [31]
*P. aridus* R2-37-08W	Wheat, USA	CP027728	Parejko et al. [27]
*P. aridus* R3-18-08	Wheat, USA	CP027729	Parejko et al. [27]
*P.* sp. LBUM920	Spruce, Canada	CP027762	Richard Hamelin
*P. yamanorum* LBUM636	Strawberry, Canada	CP012400	Morrison et al. [32]

**Table 2:** In tuber biocontrol activity of 23 PCA-producing *Pseudomonas* spp. strains against potato late blight.

Biocontrol Activity		
Weak	Intermediate	Strong
*P. aridus* R1-43-08 *P. synxantha* R2-54-08W *P. synxantha* R2-4-08W *P. synxantha* R6-28-08	*P. aridus* R3-18-08 *P. aridus* R2-37-08W *P. aridus* R2-7-07 *P. aridus* R11-23-07 *P. aridus* R4-39-08 *P. aridus* R3-S2-08 *P. aridus* R2-60-08W *P.* sp. R5-89-07 *P. orientalis* 8B *P. synxantha* 30B *P. synxantha* LBUM223	*P. yamanorum* LBUM636 *P.* sp. LBUM920 *P. aridus* R4-34-07 *P. orientalis* R4-35-08 *P. orientalis* R2-66-08W *P. orientalis* L1-3-08 *P. synxantha* 2-79

## Data Availability

The data presented in this study is contained within this article.

## Supplementary Materials

The following are available online at https://www.mdpi.com/article/10.3390/microorganisms9122525/s1, Figure S1: Photos representative of potato tuber antagonistic assays using *P. infestans* and 23 PCA-producing *Pseudomonas* spp.
