# Supplementary material for: In Tuber Biocontrol of Potato Late Blight by a Collection of Phenazine-1-Carboxylic Acid-Producing Pseudomonas spp
Source: Microorganisms. 2021 Dec 7;9(12):2525. doi: 10.3390/microorganisms9122525 (PMC8704545; doi:10.3390/microorganisms9122525)
Supplement: Supplementary file 1 [file microorganisms-09-02525-s001.zip › microorganisms-1494727-supplementary.pdf]

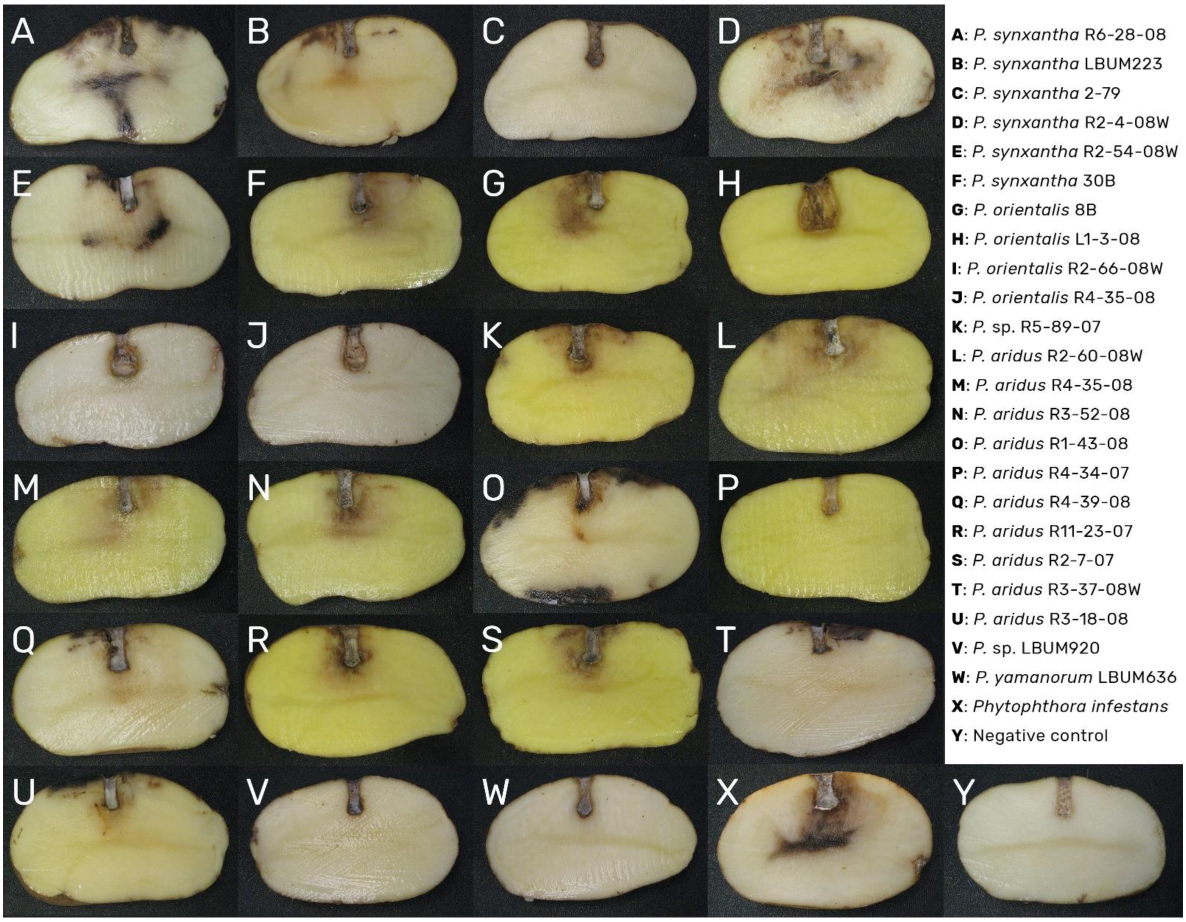

Figure S1. Photos representative of potato tuber antagonistic assays using *P. infestans* and 23 PCA-producing *Pseudomonas* spp.
